# Study of the Skull and Brain in a Cape Genet (*Genetta tigrina*) Using Computed Tomography and Magnetic Resonance Imaging

**DOI:** 10.3390/ani15233496

**Published:** 2025-12-04

**Authors:** Giuseppe Barillaro, Antonino Marcianò, Stella Costa, Matteo Marino, Simone Minniti, Claudia Dina Interlandi, Filippo Spadola

**Affiliations:** 1 San Giorgio Veterinary Clinic, 89121 Reggio Calabria, Italy; 2 Independent Resarcher, 89121 Reggio Calabria, Italy; 3 Department of Veterinary Sciences, University of Messina, 98168 Messina, Italy

**Keywords:** viverrids, cape genet, *Genetta tigrina*, magnetic resonance imaging, computed tomography, skull anatomy, brain anatomy, head, genet morphology

## Abstract

The Cape Genet (*Genetta tigrina*) is one of the exotic mammal species that are becoming popular as pets. Although it is possible to find some references in the literature regarding *Genetta* spp. and viverrids, the information about this animal remains scarce and incomplete, starting from the anatomy, a discipline that lays the foundations for deeper knowledge of a species. The aim of the present study is to identify head structures of the Cape Genet, including the skull, brain components, and the dental formula, providing for the first time a three-dimensional anatomical model of the skull, thanks to the help of advanced diagnostic imaging techniques such as computed tomography (CT) and magnetic resonance imaging (MRI), defining the ideal parameters and protocols of CT, MRI, and anesthesia for this procedure in this species. Results highlight the value of CT and MRI as study tools, enabling clear visualization of cranial structures and offering a potential reference atlas for clinicians and researchers.

## Simple Summary

Interest in exotic and unconventional animals as pets is constantly increasing, but there is paucity of literature reporting data on these animals. Specifically, there are no in-depth studies on anatomy, on which clinicians, surgeons, and radiologists, among others, can rely, and on which can lay the foundations for the study of pathology. The limited literature on the morphology of this species leads us to investigate the head components of the Cape Genet (*Genetta tigrina*) by using computed tomography and magnetic resonance imaging, to identify and report for the first time the main structures of the brain and skull of the Cape Genet (*Genetta tigrina*) and Viverrids.

## 1. Introduction

**Figure A1:**
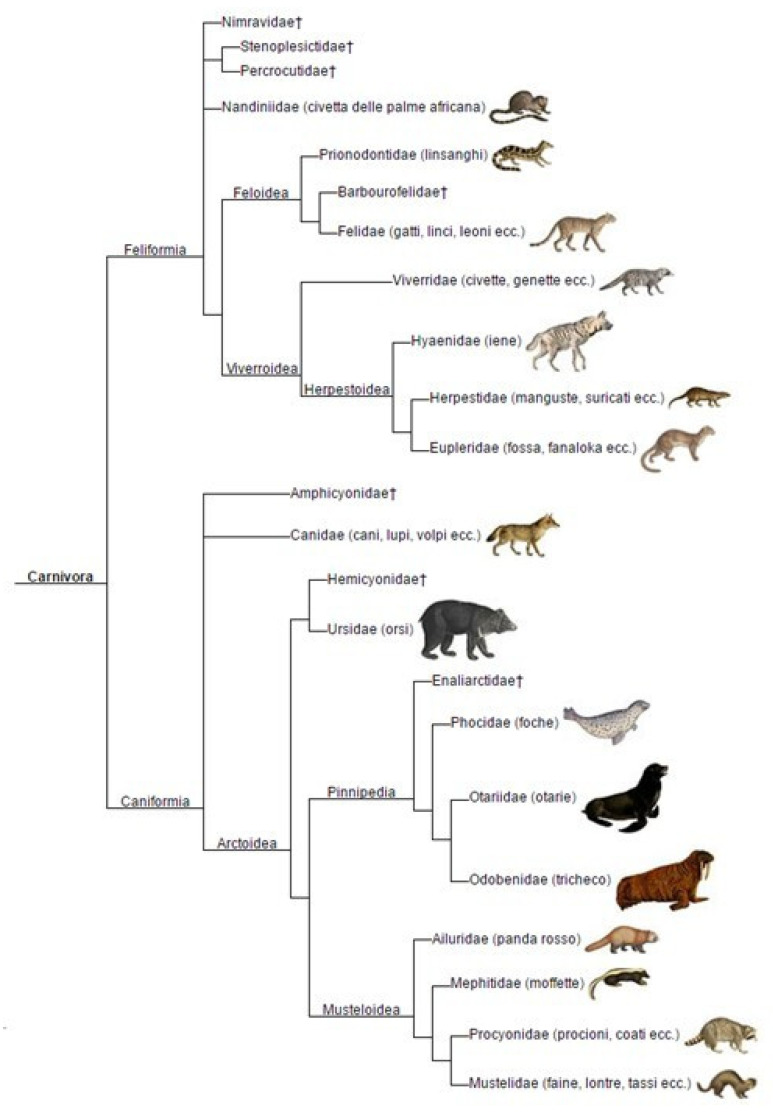
Taxonomy of mammals. From “Carnivora phylogeny”, Wikipedia, 2017 [36]. † refers to extinct species.

The Cape Genet (*Genetta tigrina*) is a small mammal belonging to the Order Carnivora, Suborder Feliformes, Family Viverridae, Genus *Genetta* (Figure A1). The distribution of this species is in South Africa, in habitats such as fynbos, grassland, and coastal forest [1]. Like many exotic species, the use of these animals as pets is spreading, similar to the meerkat and other viverrids.

The existing literature on Cape Genet to date is limited to the anatomy of the forelimb and the hindlimb of African Viverridae [2,3]. A phylogenetic study compares the skull and dentition of the currently existing *Genetta* spp. and other viverrids to the skull and dentition of their ancestors, in which the morphology of the skull is described by some measurements and images, and the dentition is reported, but a complete dental formula is not described [4]. Another study analyzes the dental form of the postcanine teeth in *Genetta* spp., again without defining a dental formula, but focusing on the shape based on the type of chewing [5]. There are other studies that deal with the skull anatomy of different Viverrids, but not the genet [6,7].

Computed tomography (CT) and magnetic resonance imaging (MRI) in exotic animals are used not only for diagnostic purposes [8], but they allow us to carry out anatomical studies as well [9]. Studies can be performed on different parts of the body such as abdomen [10], bones—even particular ones like those of birds [11]—and the head, of our interest. CT and MRI have been largely used to study the morphology of the head components (i.e., skull, brain, cranial nerves, ear, eye) of many species, exotic and not, such as dog [12,13,14], cat [15,16], Japanese Wolf [17], other canids and felids [18], horse [19,20], rabbit [21], mouse [22,23], porcupine [24,25], armadillo [26], dolphin [27,28], Atlantic Puffin [29], birds of prey [30], and iguana [31].

The aim of this study is to provide, for the first time, a detailed description and three-dimensional representation of the head anatomy of the Cape Genet (*Genetta tigrina*), including the skull, central nervous system (excluding the spinal cord, which is not located in the head), and dentition, through the use of computed tomography (CT) and magnetic resonance imaging (MRI). These imaging techniques offer innovative tools to support anatomical and clinical understanding of exotic species that are becoming increasingly common in veterinary practice, due to their increasing popularity as pets.

## 2. Materials and Methods

### 2.1. Subject Description

A 7-year-old male pet Cape Genet with neurological signs such as head tremors, ptyalism, and agitation is studied in detail.

After a general objective examination, a neurological study by CT and MR scans and an analysis of the cerebrospinal fluid was proposed to exclude intracranial pathologies. All the exams confirmed a good general state of health, which encouraged us to select this specimen for morphological study. The exams have been carried out in “San Giorgio Veterinary Clinic”, Reggio Calabria, Italy, where the owner went for a medical consultation for his pet.

### 2.2. Anesthesia

The patient was primarily sedated by an induction cage with Sevoflurane (Sevoflo, Zoetis Belgium SA, Louvain-la-Neuve, Belgium).

Meloxicam (Metacam injectable 5 mg/mL, Boehringer, Ingelheim am Rhein, Germany) 0.2 mg/kg was administered intramuscularly as an analgesic, since the procedure was not painful.

**Figure 1:**
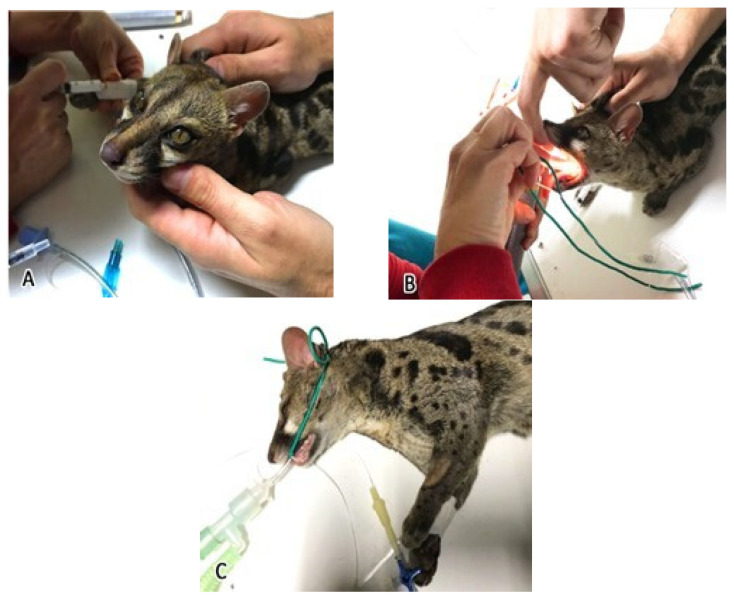
Phases of induction of anesthesia: (**A**) administration of intravenous Propofol in the cephalic vein; (**B**) insertion of the endotracheal tube with the aid of the laryngoscope; (**C**) blocking of the endotracheal tube with a rubber strap and connection to the anesthesia circuit.

A venous access was placed in the cephalic vein and, intravenously, Propofol (Propovet Multidose 10 mg/mL, Zoetis Italia S.r.l., Catania, Italy) 3 mg/kg was used for the induction phase (Figure 1A).

Afterward the animal was intubated with a cuffed endotracheal tube size 3 mm (Figure 1B,C).

Sevoflurane and oxygen were used as maintenance for the entire duration of the exams.

The animal’s vital signs were monitored (i.e., heart rate, respiratory rate, temperature, oxygen saturation, expired carbon dioxide) throughout the anesthesia period.

During this phase, it was possible to inspect the oral cavity and teeth in detail. An image of the skull was generated through an artificial intelligence system (Gemini Flash 2.5, Google, Mountain View, CA, USA, 2025) for the representation of the dental formula.

### 2.3. Computed Tomography (CT)

CT was used to exclude damage to the skull, which is considered the standard for evaluating skull structures [32].

**Figure 2:**
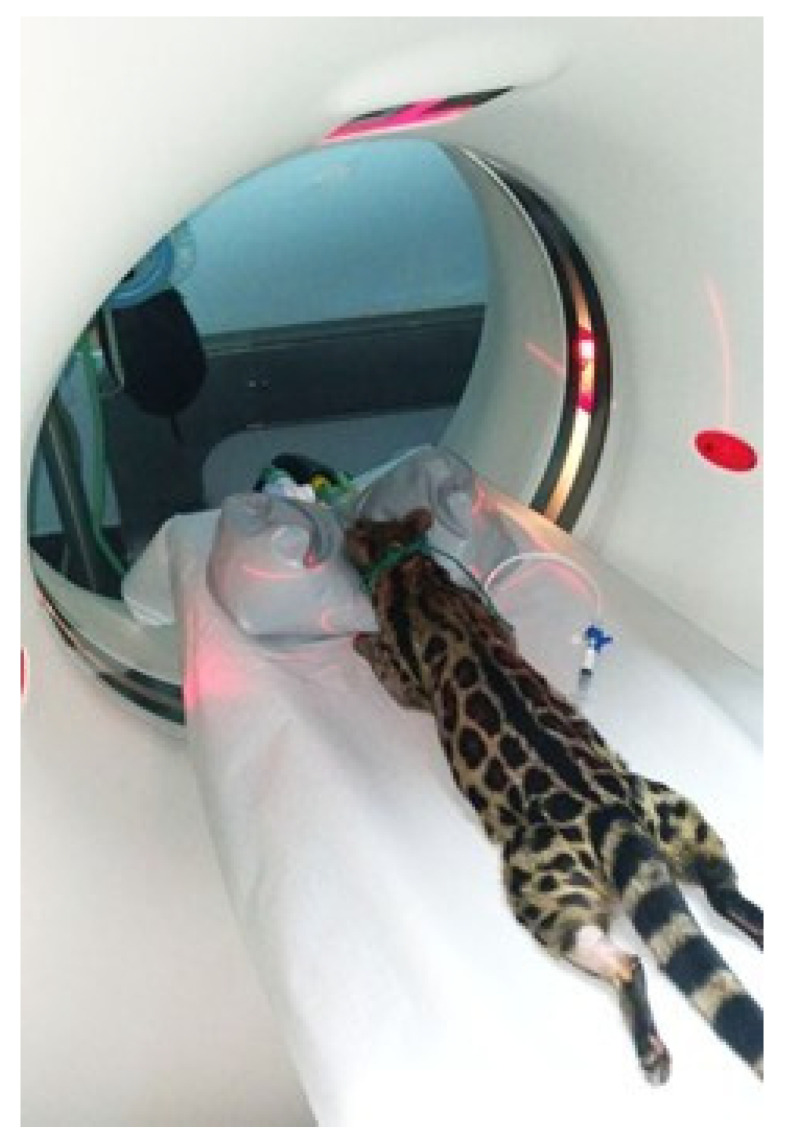
Positioning of the animal during CT examination for skull study.

The patient was positioned in sternal recumbency with the limbs in a physiological position (Figure 2).

The images were obtained with GE LightSpeed CT/e (GE Healthcare S.r.l., Milan, Italy) with a tube voltage of 120 kVp, a tube current of 80 mAs, and a slice thickness of 2.0 mm. Multiplanar reconstruction (MPR) and volumetric rendering were used to evaluate the images based on DICOM (digital imaging and communications in medicine) data, with the aid of image processing software (Horos™ Project, Geneva, Switzerland; OsiriX; v.6.0.2 64-bit, Pixmeo SARL, Bernex, Switzerland).

The MPR images have been optimized for viewing mineralized tissues, with a window level (WL) of 776 and a width (WW) of 2079. The voxel size was 0.218 mm × 0.218 mm × 2.0 mm, and the field of view (FoV) was 110.0 mm.

### 2.4. Magnetic Resonance Imaging (MRI)

**Figure 3:**
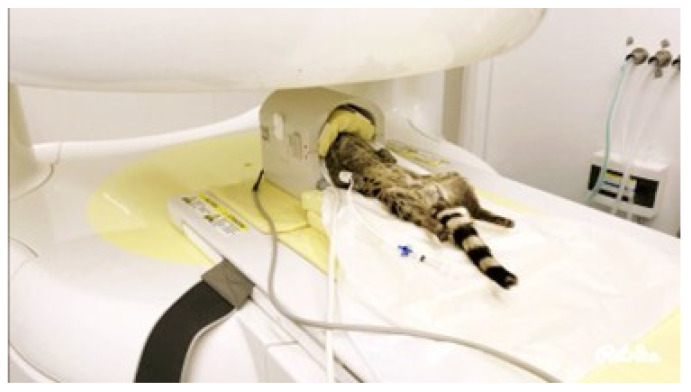
Positioning of the animal during MRI examination for brain study.

In order to search for any brain lesions not visible in CT (inflammatory/infectious lesions, neoplasms, etc.), it was considered appropriate to proceed with an MRI using a 0.3 Tesla scanner (Hitachi Airis Vento III, Fujifilm Italia SpA, Cernusco Sul Naviglio, Italy). The images were acquired using a dedicated MRI coil for the human knee. The patient was positioned in dorsal recumbency with the head and neck extended (Figure 3).

The acquisition included two-dimensional (2D) T2-weighted (T2W) images in the sagittal, transverse, and coronal planes. Two-dimensional T1-weighted (T1W) sequences were acquired in the transverse and coronal planes, while Fluid-Attenuated Inversion Recovery (FLAIR) images were obtained in the transverse plane only. The T2W images were acquired with slice thicknesses (ST) ranging from 3 to 3.5 mm, repetition times (TR) ranging from 4115 to 5860 ms, and echo times (TE) ranging from 100 to 120 ms. The 2D T1W images were acquired with ST ranging from 3 to 3.5 mm, TR ranging from 410 to 760 ms, and TE ranging from 18 to 25 ms. The FLAIR images were acquired with ST of 3.5 mm, TR of 9518 ms, and TE of 90 ms. The same image processing software that was used for the CT images was also used to evaluate these images.

By convention, the transverse images have been positioned with the left to the left of the screen and the right to the opposite side. Sagittal images were placed with the rostral portion to the left and the dorsal portion superiorly.

The parameters for image acquisition are summarized in Table 1.

## 3. Results

### 3.1. Oral Cavity

**Figure 4:**
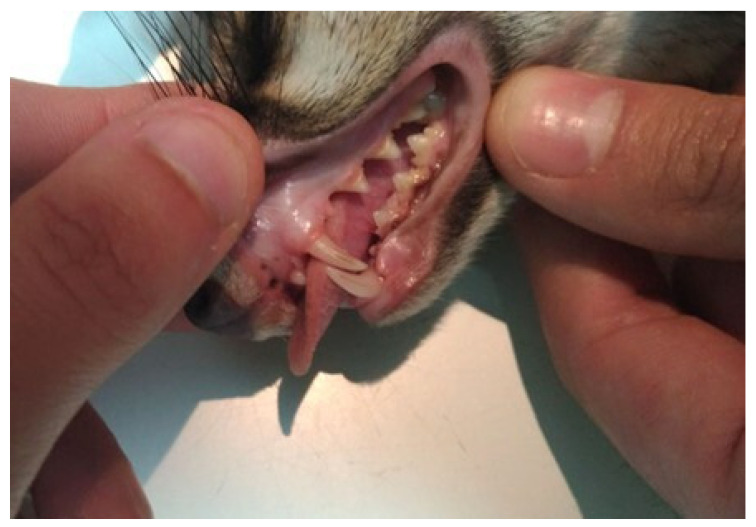
Teeth examination. All the teeth were healthy, and the dentition was complete.

The examination of the oral cavity, including anterior two thirds of the tongue, the gums, the inner surface of the cheeks and lips, the oral floor, the hard palate, and the soft palate, revealed no congenital or acquired alterations. The mucosa was physiologically colored (Figure 4).

**Figure 5:**
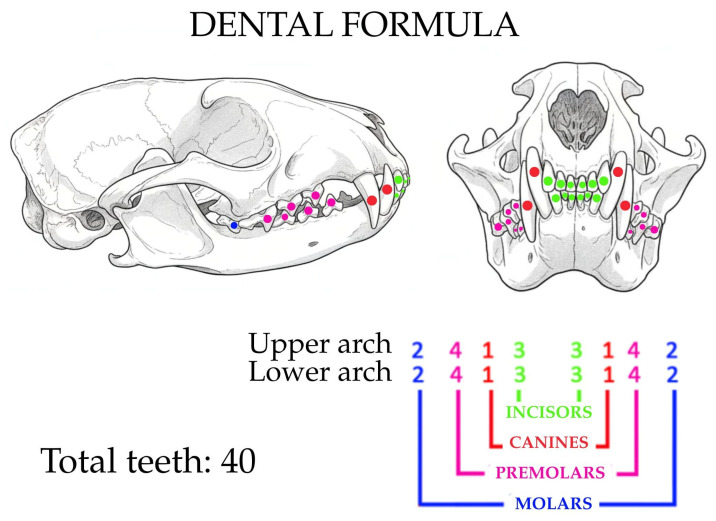
Genet dental formula. Drawing created from a photo of a *Genetta tigrina* skull with the aid of an artificial intelligence system (Gemini, Google).

All 40 teeth were intact and in good health, thus it was possible to obtain the dental formula, which is 2 (I3/3, C1/1, P4/4, M2/2) (Figure 5).

### 3.2. Computed Tomography (CT)

CT examination revealed no fractures or bone changes in the skull.

Measurements of the skull are summarized in Table 2. The length of the skull from the occipital crest to the most cranial portion of the nasal bone is 54 mm; the width of the skull measured between the two temporal bones is 31 mm; the skull thickness, taken from the temporal bone is 2 mm; the tympanic bullae measure 9 mm × 4 mm; the thickness of the tympanic bullae is 1 mm.

**Figure 6:**
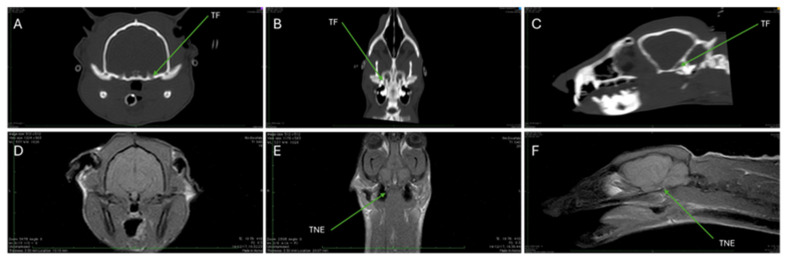
Images of the skull of a Cape Genet: (**A**–**F**). (**A**–**C**) are CT images optimized for the visualization of mineralized tissues (WL 776 and WW 2079). (**A**): Cross-section of the skull. Note the presence of the trigeminal foramen (TF) in the basicranium. (**B**): MPR post-processing in the dorsal section highlights the trigeminal foramen (TF) near the bony body of the sphenoid base. (**C**): The sagittal reconstruction highlights the trigeminal foramen (TF), which lies between the petrous part of the temporal bone and the wing of the sphenoid base. (**D**–**F**) show T1W MRI in transverse, dorsal, and sagittal sections, with TR of 410 and TE of 18. (**D**) shows a comparison of the brain. The brain, which is isointense, is located anterior to the cerebellum, below the brainstem. The muscular structures appear isointense, while the esophagus, the pharyngeal cavity, and the external auditory meatus appear hypointense. (**E**,**F**) show a hyperintense region ventrolaterally in the pontine region, attributable to trigeminal nerve emergency (TNE).

**Figure 7:**
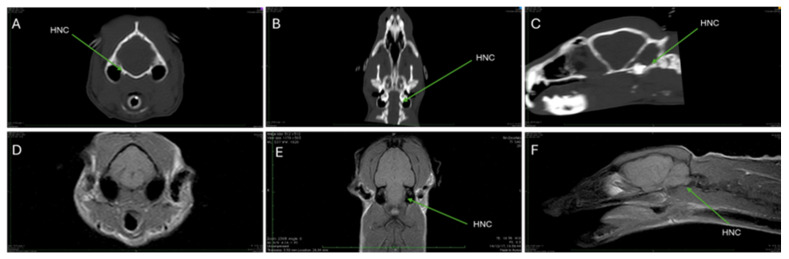
Images of the skull of a Cape Genet (**A**–**F**). (**A**–**C**): CT images optimized for visualizing mineralized tissues (WL 776 and WW 2079). (**A**): Cross-section of the skull. The hypoglossal nerve canal (HNC) in the basal part of the occipital bone is highlighted by the arrow. The canal runs adjacent to a hypodense structure: the tympanic bulla. (**B**): MPR post-processing in the dorsal section shows a deep depression in the exocranial surface of the lateral part of the occipital bone, the hypoglossal nerve canal (HNC). (**C**): The sagittal reconstruction confirms the presence of the hypoglossal nerve canal (HNC) in the basal part of the occipital bone, caudal to the temporal labyrinth, which appears hyperdense (relative to adjacent bone structures). (**D**–**F**) MRI T1W, respectively, in transverse, dorsal, and sagittal sections with TR: 410 and TE: 18. (**D**) shows the comparison with CT of the portions of the brain and muscle structures (isointense) and tympanic cavities (hypointense). (**E**): the arrow shows a hyperintense region ventrolaterally to the pontine region, which extends caudoventrally into (**F**).

Figure 6A–C and Figure 7A–C are CT images, optimized for visualizing mineralized tissues (Window Level (WL) 776 and Window Width (WW) 2079).

Figure 6A–C highlight the position of the trigeminal foramen. It is situated in the basicranium (Figure 6A, Cross-section), near the bony body of the sphenoid base (Figure 6B, Dorsal MPR Post-processing). Moreover, the trigeminal foramen lies between the petrous part of the temporal bone and the wing of the sphenoid base (Figure 6C, Sagittal Reconstruction).

Figure 7A–C focus on the location of the hypoglossal nerve canal. The hypoglossal nerve canal can be observed located in the basal part of the occipital bone. The canal is noted to run adjacent to a hypodense structure, identified as the tympanic bulla (Figure 7A, Cross-section). The hypoglossal nerve canal is visible as a deep depression on the exocranial surface (outer surface) of the lateral part of the occipital bone (Figure 7B, Dorsal MPR Post-processing). The hypoglossal nerve canal is in the basal part of the occipital bone, positioned caudal (towards the tail) to the temporal labyrinth, which appears hyperdense (brighter on CT) (Figure 7C, Sagittal Reconstruction).

**Figure 8:**
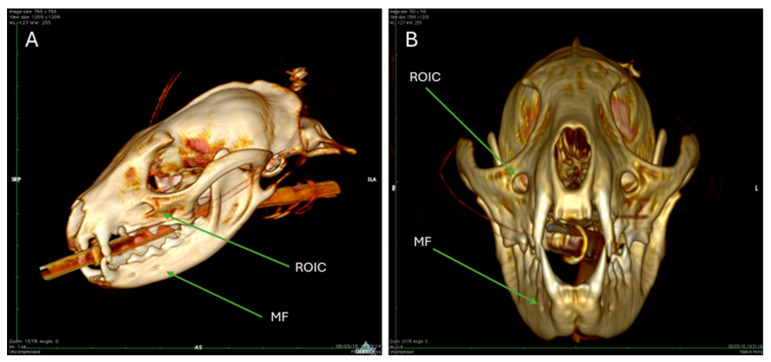
Volumetric rendering of the skull of a Cape Genet. (**A**): The 3D model illustrates the mental foramina (MF) on the lateral surface of the mandible, indicating the exit point of the inferior alveolar nerve. The rostral opening of the infraorbital canal is indicated on the maxillary bone (ROIC). (**B**): View of the mental foramina (MF) and the rostral opening of the infraorbital canal frontally through the 3D model.

Figure 8 displays a volumetric rendering (3D model) of the Cape Genet skull that illustrates the mental foramina and the rostral opening of the infraorbital canal. The mental foramina are visible on the lateral surface of the mandible. These openings represent the exit points for the terminal branches of the inferior alveolar nerve. Rostral opening of the infraorbital canal is indicated on the maxillary bone.

### 3.3. Magnetic Resonance Imaging (MRI)

As evidenced by the MRI examination, the ventricular structures appear symmetrical and normally shaped. No deviation of the sickle brain was appreciated; no areas of abnormal signal intensity were detected.

Figure 6D–F and Figure 7D–F present T1-weighted (T1W) MRI scans (TR 410, TE 18) in transverse, dorsal, and sagittal sections, focusing on soft tissues.

In Figure 6D (Transverse Comparison), the brain appears isointense and is located anterior to the cerebellum and below the brainstem; muscular structures also appear isointense, while the esophagus, pharyngeal cavity, and external auditory meatus (ear canal) appear hypointense. A hyperintense region is visible ventrolateral to the pontine region (Figure 6E,F). This specific signal is attributed to the trigeminal nerve emergency.

Figure 7D (Transverse Comparison) provides a comparison with the CT findings, showing the brain and muscle structures as isointense (similar intensity to surrounding tissue), while the tympanic cavities appear hypointense (darker). A hyperintense region positioned ventrolaterally to the pontine region is visible (Figure 7E, Dorsal Section). This hyperintense region extends caudoventrally, indicating a continuous nerve structure passing through this area (Figure 7F, Sagittal Section).

**Figure 9:**
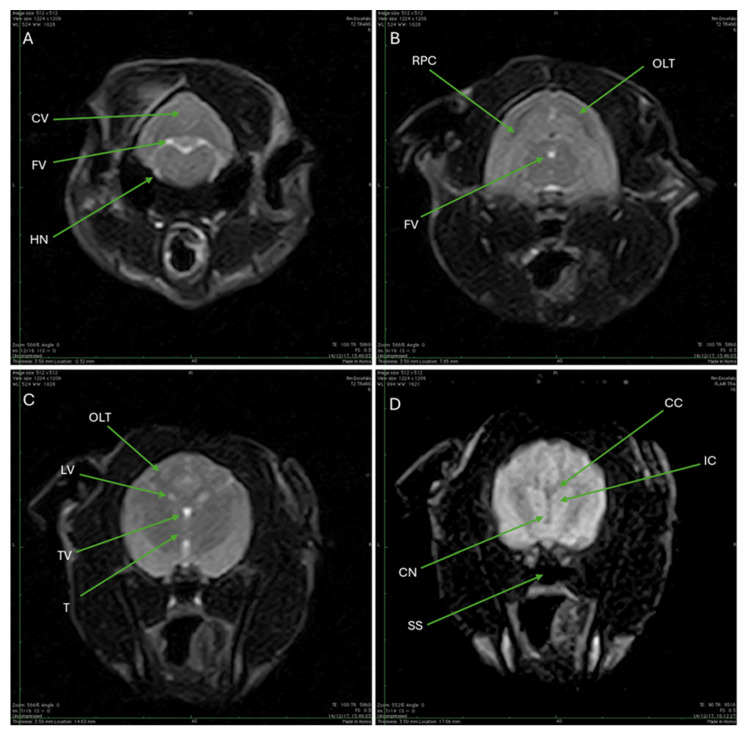
(**A**): T2W MRI (TR 5860 TE 100) shows the caudal portion of the brainstem/medulla oblongata (rhombencephalon) at the level of the foramen magnum. Superiorly (CV) is the cerebellar vermis (*cerebellar vermis*). In the central area, there is a rounded area with clear edges, which is clearly hyperintense and corresponds to the fourth ventricle (FV). Ventrally the hypoglossal nerve is visible (HN). (**B**): T2W MRI (TR 5860 TE 100) shows a transverse section of the rostral portion of the cerebellum (RPC), and the occipital lobe of the telencephalon begins to be distinguished (OLT). Centrally, marked hyperintensity due to the fluid content of the fourth ventricle is observed (FV). (**C**): T2W MRI (TR 5860 TE 100) of the cerebral hemispheres. The white matter (cortical brainstem) appears hyperintense compared to the gray matter of the brain, which is isointense (or slightly hypointense). Specifically, the occipital lobe of the telencephalon can be observed (OLT). The lateral ventricles (LV) and the third ventricle (TV) appear hyperintense compared to the cerebral substance. Inferior to the latter, the thalamus is present (T). (**D**): MR FLAIR sequence (TR 9518 TE 90). The sequence shows suppression of the cerebrospinal fluid signal, which appears hypointense. In the sequence, the corpus callosum (CC), the internal capsule (IC) and the caudate nucleus (CN) can be observed. Below the brain, a hypointense area is observed, air content within the sphenoid bone, and the sphenoid sinus (SS).

Figure 9 provides a comprehensive view of the Cape Genet’s brain structures using both T2-weighted (T2W) and Fluid-Attenuated Inversion Recovery (FLAIR) MRI sequences. The initial views are derived from the T2W sequence, characterized by a high signal from fluid (TR 5860, TE 100). The caudal portion of the brainstem, or medulla oblongata (part of the rhombencephalon), can be seen at the level of the foramen magnum, with the cerebellar vermis appearing superiorly, the fluid content of the fourth ventricle showing as a central hyperintensity, and the hypoglossal nerve visible ventrally (Figure 9A). Moving rostrally, the transverse section captures the rostral portion of the cerebellum and begins to delineate the occipital lobe of the telencephalon, while the fourth ventricle’s fluid content still presents as a marked central hyperintensity (Figure 9B). Focusing on the cerebral hemispheres, the white matter (cortical brainstem) appears hyperintense, the gray matter appears isointense or slightly hypointense; specifically, the occipital lobe of the telencephalon is observable, and the fluid-filled lateral ventricles and the third ventricle appear hyperintense relative to the surrounding cerebral substance, with the thalamus located inferior to the third ventricle (Figure 9C). The final image employs the MR FLAIR sequence (TR 9518, TE 90), which achieves suppression of the cerebrospinal fluid signal, making the CSF appear hypointense. Internal structures such as the corpus callosum, the internal capsule, and the caudate nucleus are clearly delineated; below the brain, an air cavity located within the sphenoid bone, identified as the hypointense sphenoid sinus, is also observed (Figure 9D).

**Figure 10:**
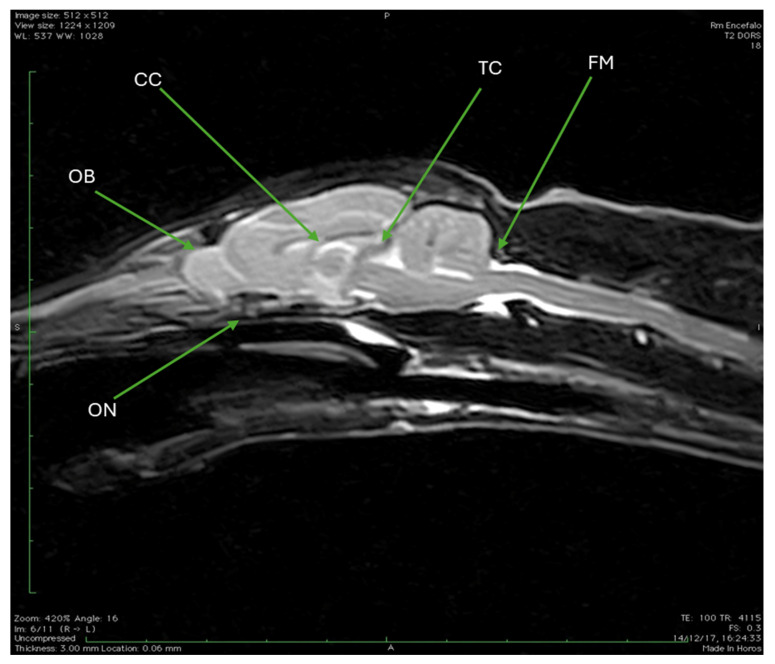
T2W MRI (TR 4115 TE 100) sagittal section. It is possible to identify the corpus callosum (CC), the olfactory bulb (OB), the first stretch of the optic nerve (ON), the tentorium cerebelli (TC), and the foramen magnum (FM).

Figure 10 presents a sagittal section acquired using a T2-weighted MRI sequence (TR 4115, TE 100), offering a midline view of the Cape Genet’s internal cranial structures. This particular image allows for the identification of several key anatomical features: superiorly, the corpus callosum is clearly visible; positioned rostrally, the olfactory bulb is distinctly observed; posteriorly, the tentorium cerebelli is delineated; the image also captures the initial segment of the optic nerve; and finally, the foramen magnum is clearly identifiable at the caudal limit of the cranial cavity.

**Figure 11:**
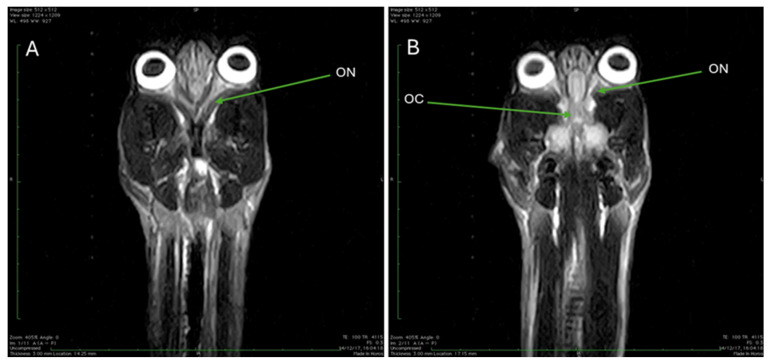
T2W MRI (TR 4115 TE 100) dorsal section. (**A**): The first stretch of the optic nerve is observed (ON). In (**B**), it is possible to identify the optic chiasm (OC).

**Figure 12:**
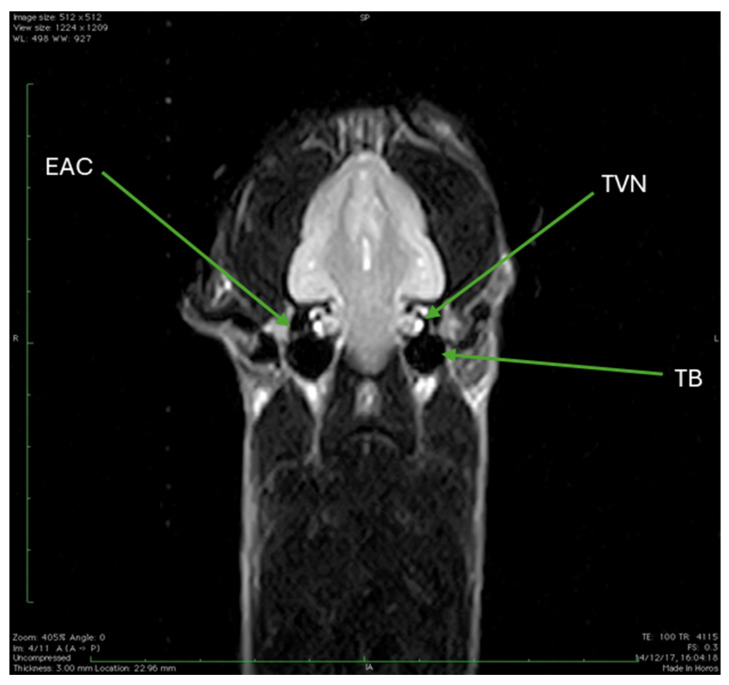
T2W MRI (TR 4115 TE 100) dorsal section. From this image, external auditory canal (EAC), trochlear vestibular nerve (TVN), and tympanic bulla (TB) can be observed.

**Figure 13:**
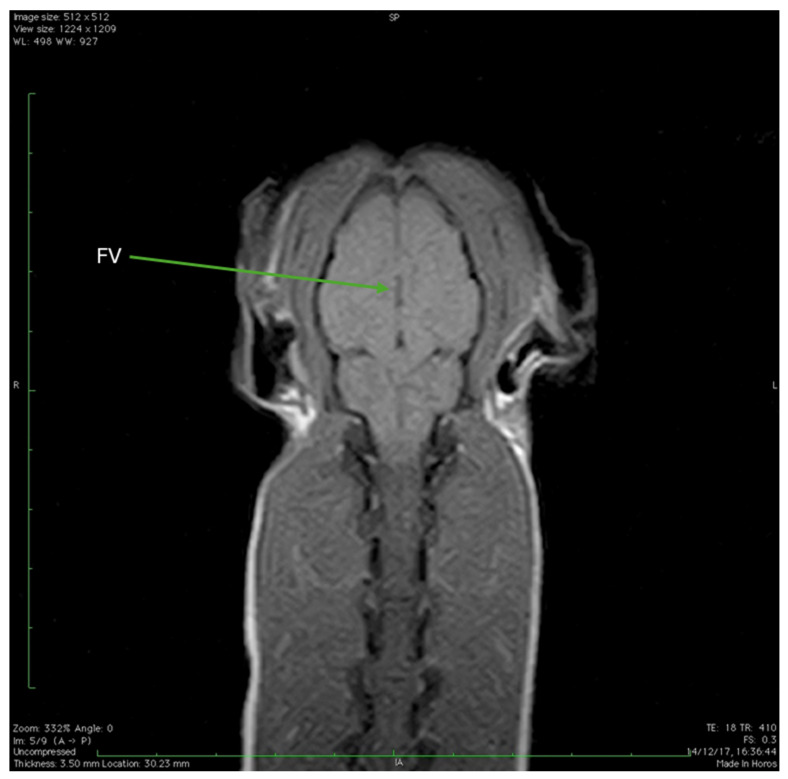
T1W MRI (TR 410 TE 18) dorsal section. From this image, fourth ventricle (FV) can be observed.

The final set of images comprises several dorsal sections obtained using a T2-weighted MRI sequence. Figure 11A,B, acquired with TR 4115 and TE 100, allow observation of the initial segment of the optic nerve, showing the optic chiasm where the two optic nerves converge and partially cross (Figure 11B). Moving to Figure 12, an image utilizing the same T2W parameters (TR 4115, TE 100) reveals structures including the external auditory canal, the trochlear vestibular nerve and the tympanic bulla. Finally, Figure 13, which uses slightly different T1W parameters (TR 410, TE 18), provides a dorsal section where the fluid-filled fourth ventricle is clearly observable.

## 4. Discussion

The Cape Genet (*Genetta tigrina*), like many other exotic animals, is a species which is increasingly common to find as a non-conventional pet, and medical interest in these animals is also increasing. To date, there are no in-depth studies on anesthesiological and pharmacological protocols, nor on the anatomy and anatomo-pathology, regarding viverrids in general, but the Cape Genet specifically [1,2,3,4,5,6,7].

Since no anesthetic protocol for this type of procedure has ever been described on this species, this study could lay the foundations for analyzing the pharmacodynamics of successfully used anesthetic drugs on this species, considering that the animal came through the procedure without any negative effects. The protocol and dosages of the drugs we used were established based on what is described for cats, as this is the phylogenetically closest species from which we could safely extrapolate data for this type of procedure. Indeed, for this procedure, deep surgical anesthesia (described in the literature for some viverrid species) was not necessary, but only simply immobilization of the animal was required, hence the choice of Propofol. Meloxicam was used only for preventive purposes related to intubation, which could be uncomfortable.

Veterinary clinicians may encounter various difficulties due to the lack of anatomical data, which implies a poor understanding of physiological appearance or pathological modifications. Thus, there is currently no basis on which veterinary clinicians can build a clinical and radiological diagnostic pathway. Morphological studies on these species are few and outdated, relying on limbs [2,3] or, of interest to us for this study, incomplete dental formulas and obsolete and equally incomplete skull studies [4,5,6,7]. These studies, however, focus more on taxonomy, classification, and differences from “allied fossil” species [1,4]. Certainly, a morphological study using CT or MRI has never been performed on this species, neither on the head nor on any other body part.

Through oral inspection, we were able to define a dental formula (Figure 5). The number of premolars and molars corresponds to that described by Gregory and Hellman (1939) for the *Genetta* spp. [4]; in addition, we identified the complete dental formula comprising incisors, canines, premolars, and molars (Figure 5). The dental formula seems to be the same as that of other viverrids, such as the Civettictis [33], Prionodon, and Poiana [7]. What varies in the dentition among the viverrid species is the shape and size, adapted to the type of diet: the genet has larger premolars and molars than species such as the binturong or the falanouc [5].

The comparative analysis of the Cape Genet’s cranial imaging, utilizing both computed tomography (CT) and magnetic resonance imaging (MRI), explicitly demonstrates the respective strengths and clinical roles of each modality in the evaluation of the skull and the central nervous system (CNS).

CT imaging excels in the assessment of bone due to its ability to optimize the visualization of mineralized tissues, as evidenced by the high-resolution images used to precisely localize bony features such as the trigeminal foramen and the hypoglossal nerve canal (Figure 6A–C and Figure 7A–C). These CT scans confirmed the absence of fractures or bone changes and allowed for the derivation of exact skull measurements, demonstrating its primary utility in assessing bone integrity and skull base architecture. Compared to the study of Gregory and Hellman (1939) [4], the total length of the skull is 2/3 compared to the measurements they reported for *Genetta* spp. (54 mm vs. 82 mm), as well as the width is almost 2/3 (31 mm vs. 50 mm). Of course, since we are only dealing with two individuals, this comparison should be taken with hesitation, without knowing the differences in the two ontogenetic maturity and sex, which could influence the results.

The 3D volumetric rendering (Figure 8) of the CT data was crucial for mapping the exit points of peripheral nerves, such as the mental foramina for the inferior alveolar nerve and the rostral opening of the infraorbital canal on the maxillary bone. While not pathological in this specimen, CT’s inherent contrast properties are invaluable for acute screening for high-density pathologies like hemorrhage and for assessing the air-filled structures, which appear hypodense, such as the tympanic bulla and the sphenoid sinus (Figure 9D) (the latter also visible on FLAIR, but confirmed by CT’s bone-optimized view).

In contrast, MRI proves vastly superior for detailed CNS assessment, owing to its exceptional soft-tissue contrast and ability to delineate specific neural structures, making it the unequivocal modality of choice for specific CNS questions like edema, demyelination, and ischemia. This superiority is confirmed by the T2W images (Figure 9A–C), which clearly distinguish white matter (hyperintense) from gray matter (isointense), a critical feature for identifying subtle neurological lesions. The visualization of deep cerebral structures like the corpus callosum, internal capsule, caudate nucleus, and thalamus on T2W and FLAIR (Figure 9C,D) highlights MRI’s unparalleled value in mapping white matter tracts and nuclei, areas where CT offers little discrimination. MRI’s sensitivity to fluid is also paramount; the marked hyperintensity of cerebrospinal fluid within the ventricles (Figure 9A–C and Figure 13) on T2W sequences demonstrates its intrinsic capability to detect edema and subtle fluid-based pathologies.

Undoubtedly, in our experience, the T1W and T2W sequences in the dorsal and transverse planes have been the most significant for the identification of the endocranial structures and for the evaluation of their symmetry.

Moreover, the use of the FLAIR sequence (Figure 9D), which achieves cerebrospinal fluid suppression, allows CNS structures near the ventricles to be viewed without signal washout, making it the gold standard for detecting early or subtle abnormalities, such as those associated with demyelination or ischemia (though no abnormal signals were detected in this healthy subject). Finally, T1W and T2W sequences (Figure 6E,F and Figure 7E,F) were instrumental in localizing a hyperintense signal in the pontine region attributed to a specific nerve structure, demonstrating MRI’s unique ability to track and visualize major soft-tissue nerves.

In summary, the combined approach provided a complete picture: CT defined the bony scaffolding and nerve exits, while the high soft-tissue contrast of MRI allowed for the comprehensive assessment of the CNS parenchyma, white matter tracts, and fluid spaces. Using CT and MRI as new tools for the study of anatomy and through the comparison with pet carnivores such as dogs and especially cats—having a morphological convergence with felids [34] (Figure A1)—it was possible to identify and describe for the first time in detail many anatomical structures of the skull and brain of the Cape Genet (*Genetta tigrina*).

This study confirms the extreme sensitivity of the advanced diagnostic techniques currently available in veterinary medicine. Their use, already widely applied to the most common animals, appears strongly indicated for exotic pets and zoo animals as well, both for clinical applications and morphological study. The images resulting from the use of these tools allowed us to study the structures under examination in detail, offering a potential reference atlas. Indeed, the clinical implications of the results we obtained concern the possibility for veterinarians to have a point of reference for this species, for which there is little literature, from a morphological, anesthesiological, and radiological standpoint. In fact, for the first time, we used CT and MRI to obtain images of both the skull and the central nervous system (with the exception of the spinal cord) of the Cape Genet. The latter, in particular, had never been described in this species or on other viverrids. What can be found to date are macroscopic descriptions of the skull and teeth, especially in other viverrid species such as Prionodon, Poiana, and Civets, where the cranial bones are well described based on the animal’s skull [5,7,33]. Regarding the *Genetta*, there are similar morphological descriptions [4], but not in detail for the *Genetta tigrina*. To describe this in a living specimen, it was essential to use imaging tools such as CT and MRI.

In a context of innovation, the use of CT and MRI is becoming increasingly popular even within universities, since they are considered “new and wonderful ways to visualize [anatomical] structures” [35], thanks to 3D visualization. This study supports and corroborates the idea that these tools can be used not only for diagnostic purposes, but also for research and study purposes.

Moreover, evaluations of the species can arise from the information obtained: as an effect of the evolutionary pressure of this efficient nocturnal predator, the dimensions of the optic nerves, eyeballs, and tympanic bullae are well developed, which makes these animals perfectly adapted to hunt in the dark, with acoustic and visual capabilities certainly out of the ordinary.

This study certainly has limitations, given the small number of animals examined (*n* = 1). Indeed, having analyzed a single animal does not allow us to compare measurements that can vary between different individuals, due to sex, age, and skeletal development. Ideally, a larger number of animals would have been available for examination, and comparisons could have been made with anatomical dissections to provide macroscopic and histological reference. Certainly, in the future, it is desirable to be able to expand the study to more animals of this species and compare these images with anatomical dissection studies, to complete the study similarly to others described in the literature, in which MRI and CT are associated with cross-sectional anatomy [10,21,25,26,27,29,31]. Having more subjects to include in the study could allow for standardization of the measurements obtained, the anesthesia protocol, and the CT and MRI parameters.

## 5. Conclusions

This study represents the first detailed anatomical and neuroanatomical investigation of the head of the Cape genet (*Genetta tigrina*), based on advanced diagnostic imaging. The use of CT and MRI enabled the in vivo assessment of both osteological and soft-tissue structures, offering high-resolution, multiplanar, and three-dimensional visualization.

This study allowed for the identification and measurement of major cranial structures, including skull morphometrics, foramina associated with cranial nerves, and key neuroanatomical landmarks of the central nervous system (with the exception of the spinal cord). The findings support the integration of CT and MRI in the diagnostic approach to exotic carnivores.

To integrate the morphological description of the head, the complete dental formula was described for the first time in this species through direct oral inspection, comparing it with the partial descriptions available for other genets and with those of other viverrids.

Furthermore, an anesthetic protocol for immobilization for diagnostic imaging procedures has been described for the first time in this species.

The volumetric data provided by this investigation can serve as a baseline for comparative anatomical studies, clinical interpretation of diagnostic images, and potential surgical or therapeutic planning. In this context, the present work may serve as an anatomical atlas and a methodological reference for veterinary professionals involved in exotic animal medicine.

Considering the limited literature available on viverrids, particularly on imaging protocols and anatomical references, this study fills an important gap in the current veterinary knowledge and provides a reproducible protocol for future evaluations in both clinical and research contexts.

Lastly, the morphological study of exotic species from a paleontological point of view is increasing. Therefore, this article could support future morphological studies for those species that have a strong phylogenetic link with some extinct species, as described by Gregory and Hellman (1939) [4].

## Figures and Tables

**Table 1:** Parameters during MR study. T2W: T2-weighted; T1W: T1-weighted; FLAIR: Fluid-Attenuated Inversion Recovery; SAG: sagittal; TRS: transverse; DOR: dorsal; RT: repeat time; ET: echo time, ST: thickness.

Sequences	Planes	RT (ms)	ET (ms)	ST (mm)
T2W	SAG	5212	120	3.0
T2W	TRS	5860	100	3.5
T2W	DOR	4115	100	3.5
T1W	SAG	700	25	3.0
T1W	TRS	760	25	3.5
T1W	DOR	410	18	3.5
FLAIR	TRA	9518	90	3.5

**Table 2:** Skull measurements.

Skull Measurements	
Length	54 mm
Max width	31 mm
Skull thickness	2 mm
Tympanic bullae	9 mm × 4 mm
Tympanic bullae thickness	1 mm

## Data Availability

The original contributions presented in this study are included in the article. Further inquiries can be directed to the corresponding author.

## Abbreviations

The following abbreviations are used in this manuscript:

CT	Computed tomography
MRI	Magnetic resonance imaging
T1W	T1-weighted
T2W	T2-weighted
T2F	T2 FLAIR
TRS	Transverse
SAG	Sagittal
DOR	Dorsal
RT/TR	Repeat time
ET/TE	Echo time
ST	Thickness
MPR	Multiplanar reconstruction
DICOM	Digital imaging and communications in medicine
WL	Window level
WW	Widht
FoV	Field of view
TF	Trigeminal foramen
TNE	Trigeminal nerve emergency
HNC	Hypoglossal nerve canal
MF	Mental foramina
ROIC	Rostral opening of the infraorbital canal
CV	Cerebellar vermis
FV	Fourth ventricle
HN	Hypoglossal nerve
RPC	Rostral portion of the cerebellum
OLT	Occipital lobe of the telencephalon
LV	Lateral ventricles
TV	Third ventricle
T	Thalamus
CC	Corpus callosum
IC	Internal capsule
CN	Caudate nucleus
SS	Sphenoid sinus
OB	Olfactory bulb
ON	Optic nerve
TC	Tentorium cerebelli
FM	Foramen magnum
OC	Optic chiasm
EAC	External auditory canal
TVN	Trochlear vestibular nerve
TB	Tympanic bulla
CNS	Central nervous system
